# White Matter Regional Volumes in Relation to Menstrual Cycle Phase and Premenstrual Dysphoric Disorder

**DOI:** 10.1016/j.bpsgos.2025.100573

**Published:** 2025-07-28

**Authors:** Elin Stenhammar, Manon Dubol, Louise Stiernman, Inger Sundström-Poromaa, Marie Bixo, Erika Comasco

**Affiliations:** a Department of Women’s and Children’s Health, SciLifeLab, Uppsala University, Uppsala, Sweden; b Department of Clinical Sciences, Umeå University, Umeå, Sweden; c Department of Women’s and Children’s Health, Uppsala University, Uppsala, Sweden

**Keywords:** Menstrual cycle, Mental health, Premenstrual dysphoric disorder, Voxel-based morphometry, White matter

## Abstract

**Background:**

Premenstrual dysphoric disorder (PMDD) is an understudied, debilitating, and hormone-related mental disorder. Recent evidence suggests alterations in white matter structure during the symptomatic luteal phase in PMDD. In this study, white matter volumes (WMVs) in the brains of women with PMDD versus control women were compared across the menstrual cycle, to determine whether these differences reflect state- or trait-like characteristics.

**Methods:**

Anatomical magnetic resonance imaging was performed during the midfollicular phase and the late luteal phase of the menstrual cycle in 28 women with PMDD and 27 control women. WMVs were assessed using voxel-based morphometry, employing both region-of-interest (ROI) and exploratory whole-brain approaches.

**Results:**

No group-by-phase interaction effects on WMVs were found. Across menstrual cycle phases, women with PMDD exhibited greater WMVs than control women within ROIs (in the bilateral uncinate fasciculus, right inferior fronto-occipital fasciculus, and left crus and fimbria of the fornix) and across the whole brain (in inferior occipital areas and near the angular gyrus), indicating trait- rather than state-like structural markers of PMDD.

**Conclusions:**

These findings suggest that women with PMDD exhibit larger WMVs than healthy control women, during both the symptomatic and asymptomatic phases of the menstrual cycle, in white matter tracts involved in emotion processing and regulation, memory, and connecting limbic and prefrontal regions of relevance to mood disorders.

## Plain Language Summary

Premenstrual dysphoric disorder (PMDD) is a severe mood disorder linked to the menstrual cycle. This study used brain scans to compare women with PMDD with healthy women at different points in their cycle. Researchers found that women with PMDD consistently had larger white matter volumes, notably in brain areas linked to mood and emotion, regardless of the symptoms. This suggests that PMDD may involve lasting brain differences, not just changes tied to hormones each month, which could help guide future treatments.

Premenstrual dysphoric disorder (PMDD) is a depressive disorder that affects 2% to 8% of menstruating women (1). Symptoms occur during the luteal phase of the menstrual cycle and comprise affective, cognitive and physical symptoms with functional impairments comparable to those seen in other depressive disorders (2,3). Aside from the relationship between symptoms and progesterone level fluctuations during the luteal phase (4,5), the pathogenesis of the disorder is not known (6), and ovarian hormone levels during the menstrual cycle do not differ between women with PMDD and healthy individuals (7,8). The current hypothesis is that PMDD symptoms are triggered by a maladaptive neural response to the changes in ovarian hormone levels (especially progesterone and its metabolite allopregnanolone) in susceptible individuals (4,9,10). Evidence suggests that PMDD may involve altered sensitivity of the GABAergic (gamma-aminobutyric acidergic) system to allopregnanolone, as well as reduced serotonergic transmission (9,11, 12, 13, 14). Notably, functional imaging studies suggest an impairment in top-down inhibitory circuits in PMDD, likely involving altered connections within the corticolimbic networks (11).

Increasing evidence indicates that ovarian hormones affect brain structure, chemistry, and function (15, 16, 17), thus potentially influencing behavior and mental health in women during their reproductive years (16,18). Given the extensive distribution of ovarian hormone receptors throughout the brain (19,20), it is plausible that neuroadaptive processes that influence brain structure and function occur concomitantly with fluctuations in ovarian hormones. For instance, changes in gray matter volume are seen in relation to the rapid estradiol and progesterone fluctuations during the menstrual cycle of healthy individuals (16,17,21). Furthermore, limited evidence suggests that white matter volumes (WMVs) in healthy, naturally cycling women show small or inconsistent variation across the menstrual cycle (22,23). Thus, failure to adapt to the hormonal changes across the menstrual cycle may provide a framework for the neural mechanisms that underpin symptom onset in PMDD.

In search of a better understanding of the pathogenesis of PMDD, magnetic resonance imaging (MRI) has been used to investigate state neuroanatomical correlates. Structural MRI research has focused on gray matter, showing smaller gray matter volumes and thinner cortices in patients with PMDD during both the symptomatic and asymptomatic phases of the menstrual cycle (24,25). Until recently, cerebral white matter in patients with PMDD remained uninvestigated (11). The first evidence of differential white matter structure associated with PMDD is based on an MRI study conducted during the symptomatic luteal phase of the menstrual cycle (26). In the Gu *et al.* (26) study, microstructural and volumetric alterations in white matter tracts that facilitate communication between cortical and subcortical limbic and paralimbic regions were identified as anatomical features of PMDD. These results are not unexpected considering that white matter alterations have been associated with affective and anxiety disorders, which share symptoms with PMDD (27). However, whether these features are only present during the symptomatic phase remains to be determined. The cyclic nature of PMDD symptomatology raises the question of whether white matter brain alterations reflect trait-like features that are specific to the disorder and independent of current hormonal status or state-like properties associated with changes in ovarian hormone levels across the menstrual cycle.

In the current study, we sought to investigate white matter structure alterations in women with PMDD compared with control women in relation to menstrual cycle phase, to determine whether differences in WMVs represent state or trait characteristics of PMDD. Participants were examined using anatomical MRI both during the asymptomatic midfollicular phase and the symptomatic late luteal phase of the menstrual cycle. Complementary region-of-interest (ROI) and whole-brain approaches were used. In this study, the fornix, cingulum, inferior fronto-occipital fasciculus, forceps minor (corpus callosum), uncinate fasciculus, anterior thalamic radiation, superior longitudinal fasciculi, and superior cerebellum peduncles were selected as ROIs, as these white matter tracts have been shown to differ in relation to affective disorders, behavioral and emotional dysregulation disorders, personality disorders, hormonal contraceptive use, and PMDD (26, 27, 28, 29, 30, 31, 32). We expected to find trait-like differences in WMVs between patients with PMDD and control participants in areas previously reported to differ between groups during the luteal phase (26), consistent with our recent report of trait-like cortical architecture alterations in PMDD (25).

## Methods and Materials

### Participants

In the context of this study, the term women is used to refer to female biological sex: specifically, individuals assigned female at birth with ovarian hormone cycles and menstrual patterns. Thirty-two participants with PMDD and 32 healthy control participants were recruited for the study by means of advertisement in local newspapers, student websites for clinical trials, social media and boards at outpatient clinics, and at the Umeå University campus in Sweden. Participants were included if they met the following criteria: overall healthy, 18 to 45 years of age, and with regular menstrual cycles (25–31 days). Women with PMDD also had to fulfill PMDD diagnostic criteria according to DSM-5. Participants were excluded if they were currently using steroid hormones (including hormonal contraceptives) and/or psychotropic or antidepressant medication and if they had significant somatic or psychiatric conditions, drug or alcohol abuse, were pregnant or had contraindications for the MRI examination. We required washout periods of 3 months for psychotropic drugs and 1 month for hormonal contraception or other hormone treatments. The participants were screened for psychiatric disorders using the Mini-International Neuropsychiatric Interview (33). Women with major depressive episodes in remission for more than 2 years prior to the study were allowed to participate. All participants completed daily ratings of premenstrual symptoms for a minimum of 2 menstrual cycles using the Daily Record of Severity of Problems (DRSP) (34), implemented via an ad hoc web platform. The study was approved by the Regional Ethical Review Board of Umeå (2016-111-31M, 2017-266-32M) and conforms to the provisions of the Declaration of Helsinki.

PMDD diagnosis was confirmed using the methodology developed by Endicott *et al.* (34) based on the following criteria: 1) daily average symptom score ≤3 (mild) during the midfollicular phase (days +6 to +10 after the onset of menses); 2) at least 2 days with ratings ≥4 (moderate) during the late luteal phase (days −5 to −1 prior to the onset of menses), for a minimum of 1 core mood symptom and at least 5 symptoms in total; and 3) symptoms during the late luteal phase interfered with daily functioning, which was defined as ratings of ≥4 for 2 days on at least 1 impairment item. If the above criteria were met for 2 consecutive menstrual cycles and agreed with the clinical judgment of the investigator, a diagnosis of PMDD was given. Women included in the control group were required to be asymptomatic across the entire menstrual cycle, i.e., no mean ratings >3 during either phase.

### Study Design

Participants underwent MRI scanning on 2 occasions, once during the midfollicular phase (menstrual cycle day +5 to +11) and once during the late luteal phase (menstrual cycle day −7 to −1). Menstrual cycle phase was confirmed by self-reported previous and next menses, as well as serum concentrations of estradiol and progesterone. Ovulation was verified based on the standard curve of day-to-day serum progesterone levels during an idealized menstrual cycle (9).

### Hormone Analysis

Serum concentrations of progesterone and estradiol were analyzed using Elecsys Gen III immunoassays at the central hospital laboratory at Norrlands University Hospital, Umeå, Sweden. Briefly, samples were first incubated with progesterone- or estradiol-specific biotinylated antibodies. Streptavidin-coated microparticles were then added together with a ruthenium complex–marked derivate for each steroid to form detectable antibody hapten complexes. Progesterone and estradiol were then quantified with chemiluminescence. Samples were then compared against a device-specific calibration curve to determine their concentrations. With this method, the detection limits were 159 pmol/L for progesterone and 18.4 pmol/L for estradiol.

### MR Acquisition

Acquisition of T1-weighted images was performed on a 3T Discovery MR750 scanner (General Electric) equipped with a 32-channel head coil, using a 3-dimensional fast spoiled gradient echo sequence (TR = 8.2 ms, TE = 3.2 ms, 512 × 512 matrix size, flip angle = 12°, 176 transversal slices, acquisition time = 8 minutes 11 seconds, voxel size = 0.48 × 0.48 × 1 mm^3^).

### Voxel-Based Morphometry

MRI data were preprocessed, quality assessed, and analyzed using the SPM12 software (Wellcome Trust Centre for Neuroimaging, University College London) implemented in MATLAB (version R2019b; The MathWorks, Inc.) and the CAT12 toolbox (https://neuro-jena.github.io/cat/), as previously described (26).

### Statistical Analyses

An a priori power analysis was run using G∗Power (35) to determine the required sample size to detect medium effects. Assuming a power (1 − β) of 0.9, and a significance rate (α) of 0.05, the required sample size to detect medium group-by-phase interaction effects was 46.

Group-by-phase interaction effects on WMV were assessed using an ROI approach based on a priori–defined regions, in which white matter structure have recently been reported to differ between women with PMDD and healthy control participants (26), to characterize affective disorders (27), and to vary in parallel with ovarian hormones during the menstrual cycle (28,36). These analyses were carried out in SPM12, based on the general linear model (GLM) using a flexible factorial design (repeated-measures analysis of variance). The design matrix was built using group (PMDD, control) as a between-subjects factor and phase (midfollicular, late luteal) and subjects as within-subjects factors. The analyses were run within bilateral anatomical masks of white matter tracts of interest: the anterior thalamic radiation, cingulum bundle, forceps minor, fornix, inferior fronto-occipital fasciculus, superior cerebellar peduncle, superior longitudinal fasciculus (SLF), and uncinate fasciculus, as defined by the HCP (Human Connectome Project) atlas.

To assess the main effect of group (across phases) and phase (across groups) on WMVs, additional 2-samples *t* tests and paired *t* tests were run. For the main analysis, these tests were run within bilateral anatomical masks of white matter tracts of interest.

Complementary to the ROI approach, we ran exploratory whole-brain voxelwise analyses to detect potential effects in brain areas that were not investigated using ROIs. The analysis was run within a mask of white matter defined by an absolute threshold set at 0.1.

Statistical significance was defined as *p* < .05, familywise error (FWE) corrected, using threshold-free cluster enhancement (TFCE) (37). A permissive visualization threshold of *p*_FWE_ < .1 was set to detect trend-level effects. To account for multiple testing across ROIs, the false discovery rate (FDR) correction was applied.

The raw volumes of the resulting significant clusters were extracted for graphical illustrations and subsequent analyses performed using SPSS version 28 (IBM Corp.). To further examine group differences within clusters identified as significant in the main analysis, follow-up analysis was performed by using a GLM in SPSS. Gamma distribution with a log link function was applied to accommodate the non-normal distribution of the dependent variable. Group was included as a fixed factor and total intracranial volume (TIV) was entered as a covariate. Associations between PMDD symptom severity and WMV were assessed by comparing late luteal DRSP scores and WMV in significant clusters.

## Results

### Participants’ Characteristics

Participants’ characteristics, menstrual cycle history, and ovarian hormones levels are presented in the Supplement (Table S1). Women with PMDD did not differ from control women in age, body mass index, TIV, psychiatric history, parity, or menstrual history. Furthermore, the MRI scans were performed at similar time points in both groups, during both the follicular and the luteal phase. As expected, serum concentrations of estradiol and progesterone did not differ between women with PMDD and control women during either the follicular or the luteal phase.

### PMDD and WMV Across the Menstrual Cycle

**Figure 1:**
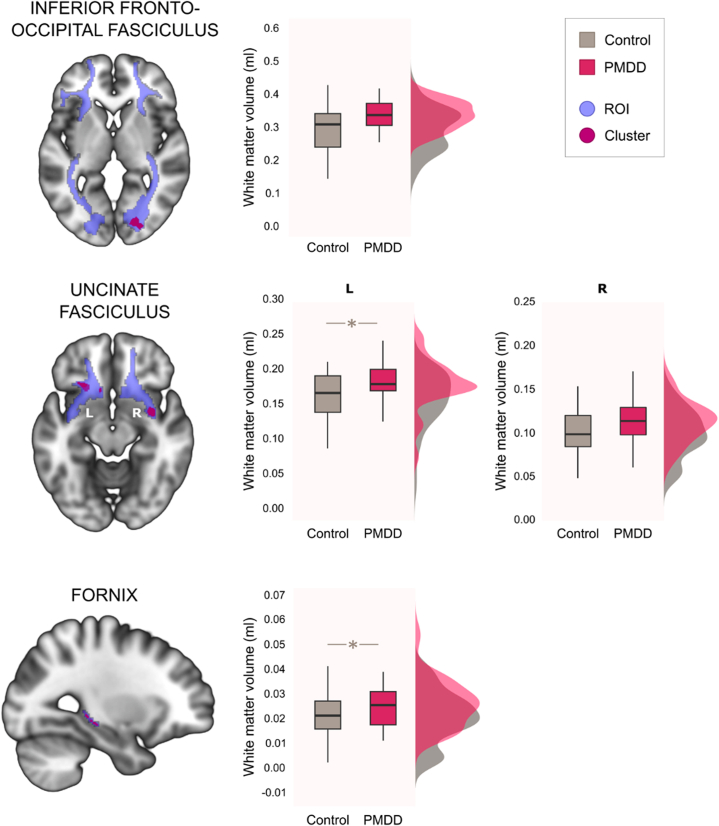
Main effect of group in regions of interest (ROIs). Volumetric white matter differences within ROIs showing clusters with greater white matter volume in the inferior fronto-occipital fasciculus, uncinate fasciculus (2 clusters, in the left [L] and right [R] hemisphere, respectively), and fornix in the premenstrual dysphoric disorder (PMDD) group compared with the control group. Results were visualized at trend level (familywise error–corrected *p* [*p*_FWE_] < .10). All peak *p* values were derived from threshold-free cluster enhancement and are presented in Table S2. ∗Survived FWE correction (*p*_FWE_ < .05). No results remained significant after false discovery rate correction.

There were no group-by-phase interactions on WMV in the predefined ROIs, indicating that there was no significant difference in WMV variation during the menstrual cycle when we compared women with PMDD with control participants. However, a significant main effect of group was found (Figure 1 and Table S2) pointing to greater volumes in women with PMDD than in control women within the bilateral uncinate fasciculus (left cluster: 88 voxels, 13.9% difference, *p*_FWE_ = .019, η_p_^2^ = 0.082; right cluster: 73 voxels, 11.7% difference, *p*_FWE_ = .067, η_p_^2^ = 0.079), the right inferior fronto-occipital fasciculus at trend level (156 voxels, 14.4% difference, *p*_FWE_ = .061, η_p_^2^ = 0.140), and the left crus/fimbria of the fornix (30 voxels, 23.4% difference, *p*_FWE_ = .028, η_p_^2^ = 0.060). However, none of the results survived FDR correction. A trend-level significant but very small main effect of phase was found, possibly indicating a false positive finding (Table S3 and Figure S1).

**Figure 2:**
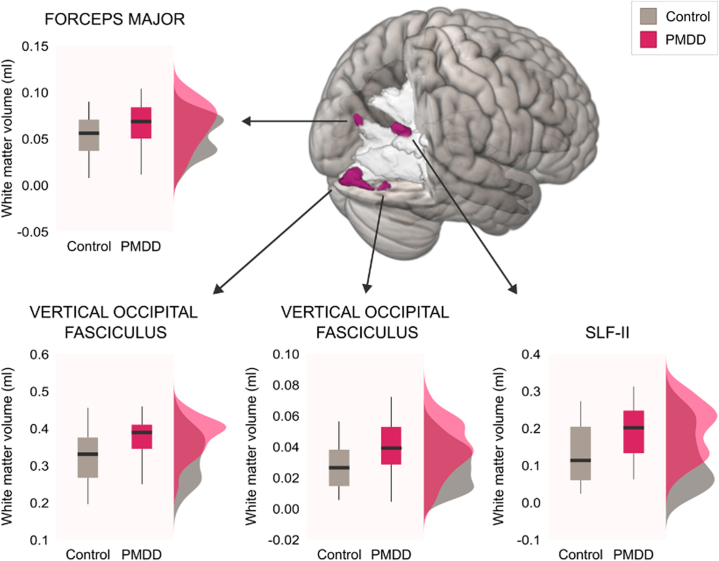
Main effect of group in the whole brain. Whole-brain volumetric white matter differences showing clusters with greater white matter volume in the forceps major, vertical occipital fasciculus (2 clusters), and second component of the superior longitudinal fasciculus (SLF-II) in the premenstrual dysphoric disorder (PMDD) group compared with the control group. All clusters are located in the right hemisphere and visualized at trend level (familywise error–corrected *p* < .10, threshold-free cluster enhancement). Peak *p* values and coordinates are presented in Table S4.

At the whole-brain level, no significant group-by-phase interaction on WMV was detected (*p*_FWE_ > .1, TFCE), consistent with the ROI findings. Also consistent with the ROI findings, a significant main effect of group was found across menstrual cycle phases, illustrating greater volumes in women with PMDD compared with healthy control women. These effects were located in inferior occipital areas and near the angular gyrus (*p*_FWE_ < .1, TFCE) (Figure 2 and Table S4). Group comparisons run on mean volumes extracted from the significant clusters in each phase confirmed that women with PMDD displayed greater WMV than control women during both the midfollicular and the late luteal phases (Table S5). No main effect of phase on WMV was observed across groups throughout the whole brain.

PMDD-related differences in volumes were not associated with symptom severity, as there was no correlation between mean total DRSP scores during the symptomatic luteal phase and mean WMV within clusters (Table S6).

## Discussion

In the current study, we used voxel-based morphometry (VBM) to characterize WMV changes across the menstrual cycle in women with PMDD compared with healthy control women. While no significant group-by-phase interaction in WMV was observed, several white matter tracts exhibited greater volumes in women with PMDD than in control women, independent of menstrual cycle phase. These findings suggest trait-like neuroanatomical alterations in PMDD and contribute to the growing body of literature on structural brain differences associated with the disorder (24,25), which potentially reflect underlying pathophysiological mechanisms.

In recent years, research on PMDD has grown substantially, raising awareness of this understudied mental disorder specific to women’s mental health. Nevertheless, the current understanding of the etiology of PMDD is tremendously limited, and knowledge of white matter alterations in PMDD is based solely on one study (26). Neuroimaging research has identified PMDD-related differences in brain function, metabolism, and neurotransmission. Interestingly, these alterations have appeared to have both state-like characteristics (fluctuating with hormonal changes) and trait-like features (persisting regardless of hormonal status) (24). While strong evidence suggests that PMDD is linked to increased sensitivity to ovarian hormone fluctuations (38), research indicates that various differences in brain structure and function may exist independently of immediate hormonal shifts (25,39, 40, 41), which is consistent with the current results. This is further supported by the lack of changes in WMV seen in response to PMDD symptom reduction through progesterone receptor modulation (42).

Consistent with results reported by Gu *et al.* (26), the uncinate fasciculus, a key structure involved in limbic-frontal communication and emotional regulation (43,44), exhibited a larger volume in women with PMDD. Therefore, altered neural structure of this tract may contribute to heightened emotional sensitivity and impaired affective control (43,44) in PMDD. Similarly, the inferior fronto-occipital fasciculus is involved in cognitive and emotional processing (45), and disruptions in this structure may therefore be associated with attentional and affective disturbances in PMDD. The involvement of the fornix, a key structure in limbic system connectivity (46), further supports the notion of disrupted emotional regulation networks in the disorder. Beyond findings in ROIs, which were defined based on evidence of white matter alterations in conditions with symptoms similar to those of PMDD (26, 27, 28, 29, 30, 31, 32), whole-brain analyses revealed greater volumes in additional WM tracts. These whole-brain clusters were located in the forceps major, vertical occipital fasciculus, and second component of the SLF. While these tracts are not part of the limbic system, growing evidence suggests that they play a supporting or indirect role in affective processes and disorders through their connectivity to broader emotional and cognitive networks (27,47). For example, disruptions in the SLF have been found in various affective disorders (27) and a previous study on PMDD (26). The observed group differences, namely greater WMV in women with PMDD compared with control participants, have been robustly demonstrated in a larger sample (26). However, given the current state of knowledge, we cannot make any assumptions about the effects or cause of these novel results.

The findings of greater WMV may seem unexpected, as psychiatric disorders generally present with lower volumes or integrity (27,48). Therefore, the larger WMV seen in this study may be disorder specific. The biological mechanisms that cause white matter changes in affective disorders are not known. Nonetheless, disruptions to white matter pathways likely reflect altered neural circuit connectivity, potentially causing affective symptoms (27). Therefore, we can only speculate on the pathophysiological processes that precipitate greater volume in specific white matter tracts. WMV differences may occur through altered numbers of axon fibers, interstitial neuronal cell plasticity, or differences in density, branching, and myelination (49). Although the literature on hormonal effects on white matter is sparse, progesterone seems to have a neuroprotective effect (50) and increase myelination in mice (51). Furthermore, estradiol peaks have been found to increase hippocampal (36) and fornix (28) white matter integrity. There is growing interest in whether inflammation plays a role in PMDD (6). However, volumetric findings related to neuroinflammation and affective disorders have been inconsistent, with several studies reporting reduced WMV, challenging the idea that inflammation might explain the current results (52, 53, 54, 55). Interestingly, allopregnanolone is expected to have an anti-inflammatory and neuroprotective effect on white matter integrity (56). However, this is unlikely to explain the current results, as we observed a main effect of group (despite both groups experiencing similar progesterone [thus also allopregnanolone] fluctuations) and an absence of a phase effect (WMV did not vary with progesterone [or allopregnanolone] levels). Nevertheless, we hypothesize that structural alterations in PMDD are either related to predisposing neurodevelopmental factors that cause vulnerability to ovarian hormone fluctuation, or are signs of maladaptive neural response to emotional brain network dysfunction, which emerges progressively with the duration of the disorder.

The current findings need to be interpreted together with methodological considerations. The greatest strength of this neuroimaging study is the longitudinal study design, where PMDD participants, for the first time, were compared with healthy control participants during different phases of the menstrual cycle in regard to WMV. In addition, menstrual cycle phase was confirmed by using both cycle tracking and hormonal assessment. Moreover, the use of prospective DRSP questionnaire scores for the selection of both PMDD and healthy control participants resulted in a reliable group comparison by ensuring that women with PMDD met DSM-5 criteria and that healthy women did not suffer from significant premenstrual symptoms consistent with undiagnosed PMDD or borderline symptomatology. However, because DRSP scores were not collected concurrently with MRI data, the absence of a correlation between PMDD symptom severity and WMV in significant clusters remains uncertain, although unlikely, given that WMV did not vary across menstrual cycle phases, which suggests independence from symptom severity. Importantly, the combination of using both whole-brain and ROI-based analyses enabled detection of both large and extended effects in the brain as an entity, as well as smaller effects in regions that are more likely affected in PMDD. Given the novel and exploratory nature of this study, a permissive threshold of *p*_FWE_ < .1 was applied to visualize trend-level effects and identify possibly meaningful patterns that merit replication in future research. While this approach increases sensitivity to potential effects in a relatively small but well-characterized sample, it also raises the possibility of false positive findings. Accordingly, findings at this threshold should be interpreted with caution and considered preliminary. Future studies with larger sample sizes and independent replication are needed to confirm the observed effects, their generalizability, and their clinical relevance. Furthermore, the study had sufficient power to detect medium effect sizes. Consequently, small effect sizes should also be assessed cautiously. Finally, structural MRI combined with VBM is limited to assessing volumetric differences. Therefore, future within-subjects studies should use diffusion tensor imaging to assess additional aspects of white matter microstructure in PMDD, such as structural connectivity or integrity.

### Conclusions

While neuroimaging studies on PMDD have largely focused on gray matter alterations, the current findings provide the first evidence of greater WMVs across the menstrual cycle in women with PMDD compared with control women. Greater volumes were found in mood-related white matter tracts during both the luteal and follicular phases of the menstrual cycle, suggesting trait- rather than state-like characteristics of PMDD. Further research is needed to determine the pathophysiological mechanisms behind the findings, their link to PMDD symptoms, and potential treatment targets.

## Supplementary Material

Figure S1 and Tables S1–S6

Key Resources Table

## References

[1] Reilly T.J., Patel S., Unachukwu I.C., Knox C.L., Wilson C.A., Craig M.C. (2024). The prevalence of premenstrual dysphoric disorder: Systematic review and meta-analysis. J Affect Disord.

[2] American Psychiatric Association (2013).

[3] Halbreich U., Borenstein J., Pearlstein T., Kahn L.S. (2003). The prevalence, impairment, impact, and burden of premenstrual dysphoric disorder (PMS/PMDD). Psychoneuroendocrinology.

[4] Schmidt P.J., Martinez P.E., Nieman L.K., Koziol D.E., Thompson K.D., Schenkel L. (2017). Premenstrual dysphoric disorder symptoms following ovarian suppression: Triggered by change in ovarian steroid levels but not continuous stable levels. Am J Psychiatry.

[5] Comasco E., Kopp Kallner H., Bixo M., Hirschberg A.L., Nyback S., de Grauw H. (2021). Ulipristal acetate for treatment of premenstrual dysphoric disorder: A proof-of-concept randomized controlled trial. Am J Psychiatry.

[6] Tiranini L., Nappi R.E. (2022). Recent advances in understanding/management of premenstrual dysphoric disorder/premenstrual syndrome. Fac Rev.

[7] Bäckström T., Sanders D., Leask R., Davidson D., Warner P., Bancroft J. (1983). Mood, sexuality, hormones, and the menstrual cycle. II. Hormone levels and their relationship to the premenstrual syndrome. Psychosom Med.

[8] Rubinow D.R., Hoban M.C., Grover G.N., Galloway D.S., Roy-Byrne P., Andersen R., Merriam G.R. (1988). Changes in plasma hormones across the menstrual cycle in patients with menstrually related mood disorder and in control subjects. Am J Obstet Gynecol.

[9] Sundström-Poromaa I., Comasco E., Sumner R., Luders E. (2020). Progesterone—Friend or foe?. Front Neuroendocrinol.

[10] Comasco E., Sundström-Poromaa I. (2015). Neuroimaging the menstrual cycle and premenstrual dysphoric disorder. Curr Psychiatry Rep.

[11] Dubol M., Epperson C.N., Lanzenberger R., Sundström-Poromaa I., Comasco E. (2020). Neuroimaging premenstrual dysphoric disorder: A systematic and critical review. Front Neuroendocrinol.

[12] Stiernman L., Comasco E., Johansson M., Bixo M. (2025). Transcription of GABAA receptor subunits in circulating monocytes and association to emotional brain function in premenstrual dysphoric disorder. Transl Psychiatry.

[13] Sundström-Poromaa I., Comasco E. (2023). New pharmacological approaches to the management of premenstrual dysphoric disorder. CNS Drugs.

[14] Sacher J., Zsido R.G., Barth C., Zientek F., Rullmann M., Luthardt J. (2023). Increase in serotonin transporter binding in patients with premenstrual dysphoric disorder across the menstrual cycle: A case-control longitudinal neuroreceptor ligand positron emission tomography imaging study. Biol Psychiatry.

[15] Barth C., Villringer A., Sacher J. (2015). Sex hormones affect neurotransmitters and shape the adult female brain during hormonal transition periods. Front Neurosci.

[16] Catenaccio E., Mu W., Lipton M.L. (2016). Estrogen- and progesterone-mediated structural neuroplasticity in women: Evidence from neuroimaging. Brain Struct Funct.

[17] Rehbein E., Hornung J., Sundström Poromaa I., Derntl B. (2021). Shaping of the female human brain by sex hormones: A review. Neuroendocrinology.

[18] Zsido R.G., Villringer A., Sacher J. (2017). Using positron emission tomography to investigate hormone-mediated neurochemical changes across the female lifespan: Implications for depression. Int Rev Psychiatry.

[19] Brinton R.D., Thompson R.F., Foy M.R., Baudry M., Wang J., Finch C.E. (2008). Progesterone receptors: Form and function in brain. Front Neuroendocrinol.

[20] Österlund M.K., Hurd Y.L. (2001). Estrogen receptors in the human forebrain and the relation to neuropsychiatric disorders. Prog Neurobiol.

[21] Dubol M., Epperson C.N., Sacher J., Pletzer B., Derntl B., Lanzenberger R. (2021). Neuroimaging the menstrual cycle: A multimodal systematic review. Front Neuroendocrinol.

[22] Meeker T.J., Veldhuijzen D.S., Keaser M.L., Gullapalli R.P., Greenspan J.D. (2020). Menstrual cycle variations in gray matter volume, white matter volume and functional connectivity: Critical impact on parietal lobe. Front Neurosci.

[23] Rizor E.J., Babenko V., Dundon N.M., Beverly-Aylwin R., Stump A., Hayes M. (2024). Menstrual cycle-driven hormone concentrations co-fluctuate with white and gray matter architecture changes across the whole brain. Hum Brain Mapp.

[24] Dubol M., Stiernman L., Wikström J., Lanzenberger R., Neill Epperson C., Sundström-Poromaa I. (2022). Differential grey matter structure in women with premenstrual dysphoric disorder: Evidence from brain morphometry and data-driven classification. Transl Psychiatry.

[25] Dubol M., Stiernman L., Sundström-Poromaa I., Bixo M., Comasco E. (2024). Cortical morphology variations during the menstrual cycle in individuals with and without premenstrual dysphoric disorder. J Affect Disord.

[26] Gu X., Dubol M., Stiernman L., Wikström J., Hahn A., Lanzenberger R. (2022). White matter microstructure and volume correlates of premenstrual dysphoric disorder. J Psychiatry Neurosci.

[27] Jenkins L.M., Barba A., Campbell M., Lamar M., Shankman S.A., Leow A.D. (2016). Shared white matter alterations across emotional disorders: A voxel-based meta-analysis of fractional anisotropy. Neuroimage Clin.

[28] De Bondt T., Van Hecke W., Veraart J., Leemans A., Sijbers J., Sunaert S. (2013). Does the use of hormonal contraceptives cause microstructural changes in cerebral white matter? Preliminary results of a DTI and tractography study. Eur Radiol.

[29] Versace A., Acuff H., Bertocci M.A., Bebko G., Almeida J.R.C., Perlman S.B. (2015). White matter structure in youth with behavioral and emotional dysregulation disorders: A probabilistic tractographic study. JAMA Psychiatry.

[30] Cyprien F., de Champfleur N.M., Deverdun J., Olié E., Le Bars E., Bonafé A. (2016). Corpus callosum integrity is affected by mood disorders and also by the suicide attempt history: A diffusion tensor imaging study. J Affect Disord.

[31] Whalley H.C., Nickson T., Pope M., Nicol K., Romaniuk L., Bastin M.E. (2015). White matter integrity and its association with affective and interpersonal symptoms in borderline personality disorder. Neuroimage Clin.

[32] Zhang R., Jiang X., Chang M., Wei S., Tang Y., Wang F. (2019). White matter abnormalities of corpus callosum in patients with bipolar disorder and suicidal ideation. Ann Gen Psychiatry.

[33] Sheehan D.V., Lecrubier Y., Sheehan K.H., Amorim P., Janavs J., Weiller E. (1998). The Mini-International Neuropsychiatric Interview (M.I.N.I.): The development and validation of a structured diagnostic psychiatric interview for DSM-IV and ICD-10. J Clin Psychiatry.

[34] Endicott J., Nee J., Harrison W. (2006). Daily Record of Severity of Problems (DRSP): Reliability and validity. Arch Womens Ment Health.

[35] Faul F., Erdfelder E., Lang A.G., Buchner A. (2007). G∗Power 3: A flexible statistical power analysis program for the social, behavioral, and biomedical sciences. Behav Res Methods.

[36] Barth C., Steele C.J., Mueller K., Rekkas V.P., Arélin K., Pampel A. (2016). In-vivo dynamics of the human hippocampus across the menstrual cycle. Sci Rep.

[37] Smith S.M., Nichols T.E. (2009). Threshold-free cluster enhancement: Addressing problems of smoothing, threshold dependence and localisation in cluster inference. Neuroimage.

[38] Rubinow D.R., Schmidt P.J. (2018). Is there a role for reproductive steroids in the etiology and treatment of affective disorders?. Dial Clin Neurosci.

[39] Stiernman L., Dubol M., Sundström-Poromaa I., Bixo M., Comasco E. (2025). Trait- versus state- grey matter volume alterations in premenstrual dysphoric disorder. https://www.researchsquare.com/article/rs-6242807/v1.

[40] Comasco E., Hahn A., Ganger S., Gingnell M., Bannbers E., Oreland L. (2014). Emotional fronto-cingulate cortex activation and brain derived neurotrophic factor polymorphism in premenstrual dysphoric disorder. Hum Brain Mapp.

[41] Dan R., Reuveni I., Canetti L., Weinstock M., Segman R., Goelman G., Bonne O. (2020). Trait-related changes in brain network topology in premenstrual dysphoric disorder. Horm Behav.

[42] Kaltsouni E., Wikström J., Lanzenberger R., Sundström-Poromaa I., Comasco E. (2024). White matter volume and treatment with selective progesterone receptor modulator in patients with premenstrual dysphoric disorder. Psychoneuroendocrinology.

[43] Xu E.P., Nguyen L., Leibenluft E., Stange J.P., Linke J.O. (2023). A meta-analysis on the uncinate fasciculus in depression. Psychol Med.

[44] Pedersen W.S., Dean D.C., Adluru N., Gresham L.K., Lee S.D., Kelly M.P. (2022). Individual variation in white matter microstructure is related to better recovery from negative stimuli. Emotion.

[45] Giampiccolo D., Herbet G., Duffau H. (2025). The inferior fronto-occipital fasciculus: Bridging phylogeny, ontogeny and functional anatomy. Brain.

[46] Lövblad K.O., Schaller K., Vargas M.I. (2014). The fornix and limbic system. Semin Ultrasound CT MR.

[47] Hodgdon E.A., Courtney K.E., Yan M., Yang R., Alam T., Walker J.C. (2022). White matter integrity in adolescent irritability: A preliminary study. Psychiatry Res Neuroimaging.

[48] Carceller-Sindreu M., Serra-Blasco M., de Diego-Adeliño J., Vives-Gilabert Y., Vicent-Gil M., Via E. (2019). Altered white matter volumes in first-episode depression: Evidence from cross-sectional and longitudinal voxel-based analyses. J Affect Disord.

[49] Zatorre R.J., Fields R.D., Johansen-Berg H. (2012). Plasticity in gray and White: Neuroimaging changes in brain structure during learning. Nat Neurosci.

[50] Guennoun R. (2020). Progesterone in the brain: Hormone, neurosteroid and neuroprotectant. Int J Mol Sci.

[51] Pletzer B., Winkler-Crepaz K., Maria Hillerer K. (2023). Progesterone and contraceptive progestin actions on the brain: A systematic review of animal studies and comparison to human neuroimaging studies. Front Neuroendocrinol.

[52] Najjar S., Pearlman D.M. (2015). Neuroinflammation and white matter pathology in schizophrenia: Systematic review. Schizophr Res.

[53] Kenk M., Selvanathan T., Rao N., Suridjan I., Rusjan P., Remington G. (2015). Imaging neuroinflammation in gray and white matter in schizophrenia: An in-vivo PET study with [18F]-FEPPA. Schizophr Bull.

[54] Najjar S., Pearlman D.M., Alper K., Najjar A., Devinsky O. (2013). Neuroinflammation and psychiatric illness. J Neuroinflammation.

[55] O’Donovan A., Bahorik A., Sidney S., Launer L.J., Yaffe K. (2021). Relationships of inflammation trajectories with white matter volume and integrity in midlife. Brain Behav Immun.

[56] Umminger L.F., Rojczyk P., Seitz-Holland J., Sollmann N., Kaufmann E., Kinzel P. (2023). White matter microstructure is associated with serum neuroactive steroids and psychological functioning. J Neurotrauma.

