# Supplementary material for: White Matter Regional Volumes in Relation to Menstrual Cycle Phase and Premenstrual Dysphoric Disorder
Source: Biol Psychiatry Glob Open Sci. 2025 Jul 28;5(6):100573. doi: 10.1016/j.bpsgos.2025.100573 (PMC12466263; doi:10.1016/j.bpsgos.2025.100573)
Supplement: Figure S1 and Tables S1–S6 [file mmc1.pdf]

SUPPLEMENTARY INFORMATION

White Matter Regional Volumes in Relation to Menstrual Cycle Phase and Premenstrual Dysphoric Disorder

Stenhammar *et al.*

Supplemental information

Table S1. Participants' characteristics, menstrual cycle history and ovarian hormones levels.

Variables	Control (n=27) n (%) or mean (± SD)	PMDD (n=28) n (%) or mean (± SD)	p-value
Age (years)	28.3 ± 5.7	28.61 ± 6.2	0.95
BMI (kg/m^2)	24.4 ± 4.1	23.65 ± 3.3	0.39
TIV (L)	1.5 ± 0.1	1.5 ± 0.1	0.28
Psychiatric history[a]			0.10
No	24 (88.8)	19 (67.9)	
Prior depression	3 (11.1)	8 (28.6)	
Prior eating disorder	0 (0.0)	1 (3.6)	
Parity			0.77
0	20 (74.1)	19 (67.9)	
≥ 1	7 (25.9)	9 (32.1)	
Menses duration (days)	5.0 ± 0.8	4.6 ± 0.7	
Menstrual cycle length (days)	28.7 ± 2.1	27.2 ± 2.0	0.09
Follicular phase assessment			
Menstrual cycle day	7.3 ± 1.3	8.6 ± 1.8	0.40
Progesterone level (nmol/L)	0.6 ± 0.3	0.7 ± 0.4	0.70
Estradiol level (pmol/L)	245 ± 201	336 ± 326	0.37
DRSP score[b]	27.1 ± 3.4	31.6 ± 9.1	0.01
Luteal phase assessment			
Menstrual cycle day	-4.0 ± 1.7	-4.4 ± 1.5	0.59
Progesterone level (nmol/L)	24.2 ± 13.7	24.3 ± 13.2	0.96
Estradiol level (pmol/L)	413 ± 183	446 ± 226	0.80
DRSP score[b]	27.8 ± 3.2	73.3 ± 16.4	< 0.01

Statistical analyses by Chi-square tests, independent t-tests or Mann-Whitney U-test. Abbreviations: BMI = body mass index, DRSP = Daily Record of Severity of Problems, PMDD = premenstrual dysphoric disorder, TIV = total intracranial volume.

[a] Psychiatric history is based on self-reports.

[b] Mean daily DRSP score during the mid-follicular (menstrual cycle day 5 to 9) or late luteal phase (menstrual cycle day -5 to -1), respectively. DRSP scores were collected during two consecutive menstrual cycles before the first magnetic resonance imaging scan.

Table S2: Main effect of group on white matter volume in regions of interest.

Region of Interest	Cluster size (voxels)	p_{FWE} (TFCE)	MNI coordinates x	y	z	η_p^2
**PMDD > Controls**						
Anterior Thalamic radiations			N.S.			
Cingulum bundle			N.S.			
Corpus callosum (Forceps minor)			N.S.			
Fornix	30	0.028	-21	-36	5	0.060
		0.033	-24	-36	2	
		0.063	-27	-26	-11	
		0.072	-27	-35	-3	
Inferior fronto-occipital fasciculus	*156*	*0.061*	*20*	*-81*	*0*	*0.140*
		0.063	*17*	*-86*	*-5*	
		0.073	*27*	*-87*	*0*	
Superior cerebellar peduncle			N.S.			
Superior longitudinal fasciculus			N.S.			
Uncinate fasciculus	88	0.019	-23	21	-17	0.082
		0.027	-20	18	-18	
		0.035	-29	26	-14	
	73	*0.067*	*30*	*2*	*-11*	*0.079*
		0.069	*33*	*0*	*-15*	
**Controls > PMDD**			N.S.			

All peak p-values are derived from Threshold-Free Cluster Enhancement (TFCE). Trend-level results were visualized at a threshold of $p_{FWE} < 0.10$ and are shown in italic. No results remained significant after False Discovery Rate (FDR) correction. Abbreviations: FWE = Family Wise Error correction, MNI = Montreal Neurological Institute, N.S. = Non-significant, PMDD = premenstrual dysphoric disorder, TFCE = Threshold Free Cluster Enhancement.

Table S3: Main effect of phase on white matter volume in regions of interest.

Region of Interest	Cluster size (voxels)	p_{FWE} (TFCE)	MNI coordinates x	y	z	η_p^2
**Mid-follicular > Late luteal**						
Anterior Thalamic radiations	53	*0.079*<br>*0.080*	*-14*<br>*-10*	*-3*<br>*-12*	*4*<br>*0*	*0.044*
Cingulum bundle		N.S.				
Corpus callosum (Forceps minor)		N.S.				
Fornix		N.S.				
Inferior fronto-occipital fasciculus		N.S.				
Superior cerebellar peduncle	42	*0.058*<br>*0.064*<br>*0.071*	*-15*<br>*-16*<br>*-14*	*-6*<br>*-10*<br>*-12*	*3*<br>*3*<br>*0*	*0.111*
Superior longitudinal fasciculus		N.S.				
Uncinate fasciculus		N.S.				
**Mid-follicular < Late luteal**						
All ROIs		N.S.				

Trend-level results were visualized at a threshold of $p_{FWE} < 0.10$ and are shown in italic. Abbreviations: FWE = Family Wise Error correction, MNI = Montreal Neurological Institute, N.S. = Non-significant, PMDD = premenstrual dysphoric disorder, TFCE = Threshold Free Cluster Enhancement.

Table S4: Main effect of group on white matter volume over the whole brain, across menstrual cycle phases.

Direction	Cluster size (voxels)	p_{FWE}	MNI coordinates x	y	z	Nearest gray matter (AAL)	White matter tract (XTRACT)	η_p^2
PMDD > Controls	127	0.067	44	-68	32	Angular, R	SLF II, R	0.170
PMDD > Controls	244	0.070	36	-89	-8	Occipital Inf, R	VOF, R	0.201
PMDD > Controls	39	0.091	15	-84	27	Cuneus, R	Forceps Major, R	0.070
PMDD > Controls	26	0.093	42	-77	-8	Occipital Inf, R	VOF, R	0.133
		0.098	45	-74	-6	Temporal Inf, R		

All peak p-values are derived from Threshold-Free Cluster Enhancement (TFCE). Redundant peaks inside clusters are not listed. The XTRACT atlas was used to extract cluster locations within white matter tracts. Abbreviations: AAL = Automatic Anatomical Labelling atlas, FWE = Family Wise Error correction, Inf = inferior, MNI = Montreal Neurological Institute, PMDD = premenstrual dysphoric disorder, R = right, SLF II = second component of the superior longitudinal fasciculus, VOF = vertical occipital fasciculus.

Table S5: Additional group comparison of white matter volume in each menstrual cycle phase, in significant clusters showing a main effect of group.

Cluster	Mid-follicular phase			Late luteal phase		
	X^2	p	Cohen's d	X^2	p	Cohen's d
**PMDD > Controls, ROI findings**						
Uncinate fasciculus, 88 voxels	2.577	0.108	-0.594	2.105	0.147	-0.578
Uncinate fasciculus, 73 voxels	1.488	0.222	-0.417	5.338	0.021*	-0.644
Fornix, 30 voxels	1.135	0.287	-0.385	2.608	0.106	-0.585
Inferior fronto-occipital fasciculus, 156 voxels	7.127	0.008*	-0.795	6.300	0.012*	-0.774
**PMDD > Controls, Whole-brain findings**						
NA[a], 127 voxels	7.693	0.006*	-0.891	7.406	0.007*	-0.884
Vertical occipital fasciculus (right), 244 voxels	10.524	0.001*	-0.946	10.180	0.001*	-0.920
Forceps major (right hemisphere projections), 39 voxels	2.676	0.102	-0.573	2.224	0.136	-0.492
Vertical occipital fasciculus (right), 26 voxels	5.953	0.015*	-0.751	6.265	0.012*	-0.773

Group differences in WMV for significant clusters, analyzed using a Generalized Linear Model (Gamma distribution, log link), with group as a fixed factor and total intracranial volume as covariate. Reported are Wald Chi-square (X^2)-values, corresponding p-values and effect sizes (Cohen's d). Cluster locations were based on overlay with atlas based on the Human Connectome Project. FWE-corrected, TFCE. Abbreviations: FWE = Family-Wise Error corrected, PMDD = premenstrual dysphoric disorder, ROI = regions of interest, TFCE= Threshold-Free Cluster Enhancement.

[a] Non-attributional. The closest region was mid-Occipital (right)
*Survived False Discovery Rate (FDR) correction for multiple testing across tested clusters (FDR corrected p-value <0.05).

Table S6: Correlation between PMDD symptom severity and white matter volume in the luteal phase.

Cluster	r	p
Uncinate fasciculus, 88 voxels	-0.14	0.46
Uncinate fasciculus, 73 voxels	0.20	0.32
Fornix, 30 voxels	0.18	0.36
Inferior fronto-occipital fasciculus, 156 voxels	0.05	0.81

2-tailed significance (p) and Pearson correlation coefficient (r) for the correlation between mean total Daily Record of Severity of Problems (DRSP) scores in the premenstrual dysphoric disorder (PMDD) group during the late luteal phase and mean white matter volumes (WMV) for each cluster, averaged over the mid-follicular and late luteal timepoints. Correlations between mean total DRSP score difference between the follicular and luteal phase and mean WMV within clusters were additionally tested without any significant results and are not presented. DRSP scores were collected during two consecutive menstrual cycles before the first magnetic resonance imaging scan.

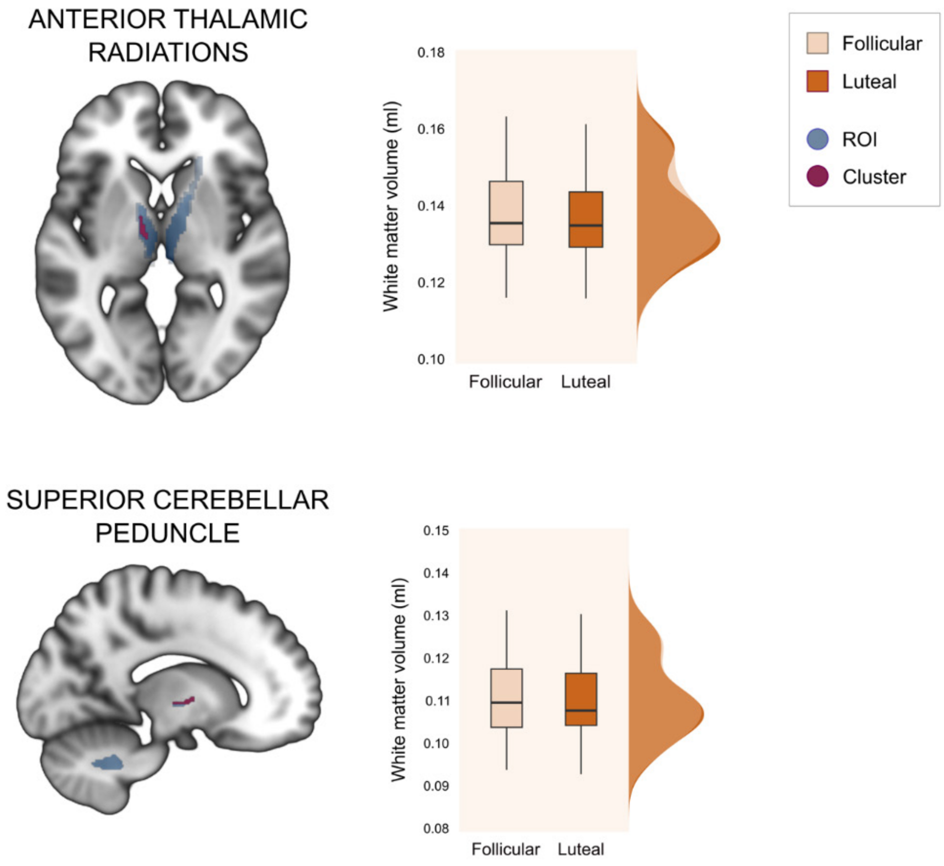

Figure S1. Main effect of phase in regions of interest. Volumetric white matter differences within regions of interest, showing two clusters with a minimal decrease in white matter volume in the late-luteal phase compared to the mid-follicular phase, in both diagnostic groups (PMDD and control), visualized at trend level ($p_{FWE} < 0.10$). All peak p-values are derived from Threshold-Free Cluster Enhancement (TFCE) and are presented in the supplement, Table S3. Follicular = mid-follicular phase, FWE = Family Wise Error corrected, Luteal = late luteal phase, PMDD = premenstrual dysphoric disorder, ROI = region of interest.
